# Polyphenic risk score shows robust predictive ability for long-term future suicidality

**DOI:** 10.1007/s44192-022-00016-z

**Published:** 2022-06-13

**Authors:** M. Cheng, K. Roseberry, Y. Choi, L. Quast, M. Gaines, G. Sandusky, J. A. Kline, P. Bogdan, A. B. Niculescu

**Affiliations:** 1 Ming Hsieh Department of Electrical and Computer Engineering, University of Southern California, 3740 McClintock Avenue, EEB 304, Los Angeles, CA USA; 2 Department of Psychiatry, Indiana University School of Medicine, Neuroscience Research Building 200B, 320 W. 15th Street, Indianapolis, IN 46202 USA; 3 Department of Pathology, Indiana University School of Medicine, Indianapolis, USA; 4 Department of Emergency Medicine, Indiana University School of Medicine, Indianapolis, USA; 5 Present Address: Department of Emergency Medicine, Wayne State University, Detroit, MI USA; 6 Stark Neuroscience Research Institute, Indiana University School of Medicine, Indianapolis, IN USA; 7 Indianapolis VA Medical Center, Indianapolis, IN USA

**Keywords:** Suicidality, Emergency department, Risk, Prediction, Machine learning, Social Isolation

## Abstract

Suicides are preventable tragedies, if risk factors are tracked and mitigated. We had previously developed a new quantitative suicidality risk assessment instrument (Convergent Functional Information for Suicidality, CFI-S), which is in essence a simple polyphenic risk score, and deployed it in a busy urban hospital Emergency Department, in a naturalistic cohort of consecutive patients. We report a four years follow-up of that population (n = 482). Overall, the single administration of the CFI-S was significantly predictive of suicidality over the ensuing 4 years (occurrence- ROC AUC 80%, severity- Pearson correlation 0.44, imminence-Cox regression Hazard Ratio 1.33). The best predictive single phenes (phenotypic items) were feeling useless (not needed), a past history of suicidality, and social isolation. We next used machine learning approaches to enhance the predictive ability of CFI-S. We divided the population into a discovery cohort (n = 255) and testing cohort (n = 227), and developed a deep neural network algorithm that showed increased accuracy for predicting risk of future suicidality (increasing the ROC AUC from 80 to 90%), as well as a similarity network classifier for visualizing patient’s risk. We propose that the widespread use of CFI-S for screening purposes, with or without machine learning enhancements, can boost suicidality prevention efforts. This study also identified as top risk factors for suicidality addressable social determinants.

**Supplementary Information:**

The online version contains supplementary material available at 10.1007/s44192-022-00016-z.

## Introduction

“It's tough to make predictions, especially about the future.”

Yogi Berra

One person dies by suicide every 40 s worldwide. Suicides are preventable tragedies. The social, psychological and biological risk factors are increasingly being known [1–3]. What has been missing in practice has been a quantitative tool that identifies this risk, provides actionable information, and pivots to a personalized treatment plan. Similar to genetics, where polygenic risk scores can provide some predictive ability, we have developed in recent years for suicidality both polygenic risk scores (based on blood biomarker transcriptomic data), and a polyphenic risk score (based on phenotypic data, i.e., known risk factors for suicidality). The latter was described as an instrument, Convergent Functional Information for Suicidality, CFI-S, which was used in veterans [1], as well as a civilian population [4]. The civilian study was a naturalistic study in a cohort of all-comers to a busy urban Emergency Department, (of Eskenazi Health hospital, the major safety net hospital in Indianapolis, IN). The patients received the standard 2-item suicide screening question, were seen by an attending physician who was asked to fill a VAS scale (visual analog scale, based on physician gestalt) about likelihood of future suicidality (ideation, planning, attempts, hospitalizations) in subsequent 6 months, and received a paper version of the CFI-S. Of note, the CFI-S does not ask about suicidal ideation. At 6 months follow-up, the CFI-S was more predictive of suicidality than the other two assessments, particularly in women [4]. An important issue to be addressed was its ability to predict long-term risk. We describe a 4-year follow-up of that population, and analyses using state-of the art machine learning approaches.

## Materials and methods

### Cohorts

Participants were enrolled in 2016-2017 in the study “Assessing Risk of Future Suicidality in Emergency Department Patients” 4. The original study was reviewed and approved by the Indiana University School of Medicine Institutional Review Board (IRB) (study number 2012150820). All participants signed informed consents, which included consent to be followed-up. This study was conducted as a follow-up study, separate ethical approval was waived by the Indiana University School of Medicine Institutional Review Board (IRB) in view of the EMR retrospective nature of the study. All follow-up was done via electronic medical record (EMR) review rather than direct patient interview. Thus data related to suicidality was obtained from EMR-documented outpatient visits and emergency department visits, where patients were assessed at that time and triaged by mental health and/or emergency department providers if showing signs of suicidality, using the current standard of care. The original study was reviewed and approved by the Indiana University School of Medicine Institutional Review Board (IRB) (study number 2012150820). All participants signed informed consents, which included consent to be followed-up. This study was conducted as a follow-up study, separate ethical approval was waived by the Indiana University School of Medicine Institutional Review Board (IRB) in view of the EMR retrospective nature of the study.

The electronic medical record was reviewed by 3 psychiatrists independently. Scores for suicidality (suicidal ideation, suicidal planning, suicide attempt, hospitalization due to suicidality) were compared between the different scorers, with looking at all notes in the EMR that had been entered since each participant was initially enrolled in the first study in 2019 (with the first enrollment of patients being 6/17/2016). Following this the scores were normalized by time through finding the shortest length of time between enrollment date and chart access, and eliminating any scores performed at a longer duration than that shortest follow-up date.

In addition to review of the EMR to gather information regarding suicidality, the Marion County Coroner’s Office database was also searched. None of the participants were found to have died by suicide locally.

### Traditional analyses

Following the collection of data a few separate analyses were performed. First, a Receiver Operating Curve Area Under the Curve (ROC AUC) for predicting future suicidality was calculated, for the 4-year follow-up. Second, a t-test was done comparing the CFI-S score between those with suicidality compared to those who did not have any over the 4 year follow-up. The analysis was also conducted at an individual item level. Third, a Pearson correlation analysis was done to determine how the CFI-S scores compare to the severity of suicidality. Suicidality was rated by severity in the following manner: those with suicidal ideation only received a score of 1, those with a plan received a score of 2, those with suicide attempt(s) received a score of 3, and those with hospitalization(s) for suicidality received a score of 4. Evaluating suicidality as a spectrum is supported by our previous work [1, 5, 6], which is consistent also with suicidality being its own free-standing diagnostic entity [7]. Fourth, a Cox-regression was performed, looking at the Hazard Ratio for the CFI-S score predicting future suicidality.

### Machine learning analyses

We performed a machine learning-based suicidality classification using the CFI-S records as input. Each patient has a CFI-S record, e.g., answers to 22 yes/no questions. The input or feature of our machine learning models are vectors with length 22 + 3, i.e., 22 yes/no answers, total number of yes, total number of answers, and the CFI-S score. The experiment is designed as follows: for input data, we use the afore-mentioned features to feed in machine learning models, and the suicidality results (ideation, planning, attempt, hospitalization) are converted to binary indicators to represent if a patient has suicidality or not. Therefore, to solve this suicidality classification problem we make use of five machine learning models, naive Bayes (NB) [8], XGBoost (XGB) [9], random forest (RF) [10], support vector machines (SVM) [11], and deep neural network (DNN) [12] classifiers. We train and tune the hyper-parameters of our machine learning models with the discovery cohort, which is commonly accepted as the training set in the machine learning field. We then fix the model and hyperparameters and test on an independent test cohort, which is referred to as the test set in machine learning. The results of the test cohort reveal better the generalizability of our approach. In this classification problem, we used AUROC, accuracy, precision, recall, and F1-score as evaluation metrics to comprehensively compare and evaluate our models. Detailed formulas for evaluation metrics are described in Supplementary Materials.

In the binary classification of suicidality, we would like to predict if a patient will have or not any suicidality. In what follows, we further inspect those patients who have suicidality, and we predict two tasks: (i) how soon the patients would take such actions (imminence prediction), and (ii) how severe the behavior would be (severity prediction).

In imminence prediction, we take the first time (in terms of month) of actions as imminence labels, and the input features are the same as in suicidality classification. For example, if a patient has suicidal ideation recorded 1 month after she/he take the CFI-S test, a suicide attempt recorded 3 months after, and a hospitalization recorded 4 months after, we take the earliest record and label the imminence as 1 month.

In severity prediction, we take weighted severity as labels, i.e., severity is composed of four parts: ideation (SI), planning (SP), attempt (SA), and hospitalization (HP), and they weigh differently based on the severity of the action. More specifically, SI = 1, SP = 2, SA = 3, and HP = 4. Different from suicidality classification, imminence and severity prediction are regression problems, and we use DNNs for prediction and evaluate it by accuracy with prediction interval (PI), root mean squared error (RMSE), mean absolute error (MAE), R-squared, and standard deviation.

The accuracy with PI is used to calculate the accuracy for our regression problem. Assume we have a data point with feature *x* and ground truth label *y*, DNN takes *x* as input and predicts the output value *y*’. Then, the actuarial *prediction* made by DNN model is an interval: [*y*’-PI, *y*’ + PI], where PI is prediction interval calculated as z*stdev (*z* takes values ranging from [1.15, 2.58] for 75% to 99% PI, and *stdev* is the standard deviation of *y*’s).

#### Deep neural networks’ hyperparameters and training details

The DNN we used in suicidality prediction is designed for classification with about 400 k parameters. The input layer has 25 neurons and there are two hidden layers with 64 neurons each. Each fully connected hidden layer is followed by a dropout layer with 50% keep rate and the ReLU [13] activation function is applied to all hidden layers. We followed standard deep neural network hyperparameter tuning methods and determined that a neural network with 400 k parameters is a decent model for this binary classification problem. Dropout is introduced to avoid overfitting. The output layer has 2 output neurons with the Softmax [14] activation function being used as the activation function. We use Adam [15] optimizer with 0.001 learning rate and binary cross entropy loss function. We split the discovery cohort (255 samples) as a training (80%) and an evaluation (20%) set for hyperparameter tuning, and the discovery results reported are evaluated on the evaluation set. After hyperparameter tuning, we train the DNN with the whole discovery cohort and lock it for testing on the test cohort (227 samples). Other machine learning models’ hyperparameter tuning, training and test follow exactly the same procedure for fair comparison.

The DNN we used in imminence and severity prediction is designed for regression. The input layer has 25 neurons, and there are 2 hidden layers. The first hidden layer is a fully connected layer with 32 hidden neurons and Sigmoid [16] activation function. The second hidden layer is a fully connected layer with 64 hidden neurons and ReLU activation function. Both hidden layers are followed by a dropout layer with 50% keep rate. The final output layer contains only 1 output neuron and a ReLU activation function is applied to rectify the output to a non-negative value. We follow standard deep neural network hyperparameter tuning and use Adam optimizer with 0.001 learning rate and a mean squared error loss function. In severity and imminence prediction, we take those samples with suicidality = 1 and this results in 56 samples selected from the discovery cohort and 50 samples selected from the test cohort. The hyperparameter tuning, training and test for all machine learning models in imminence and severity prediction follow the same procedure as in suicidality prediction.

#### Network representation

We form a similarity network of all patients based on their CFI-S records. In this network, each node represents a patient, and the link between two nodes (patients) are the similarity between these two nodes. With the answers to the questionnaire, each patient is described by a vector of size 22 + 3, where *yes* and *no* are taken as 1 and 0, other answers are taken as -1. With these vectors, we calculate the cosine similarity between vectors as the weight between two nodes. With node representation and weights, we construct an all-to-all network first, and then delete connections between nodes according to a threshold in the edge’ weights, i.e., if the weights between two nodes are less than a threshold (0.995), we delete that link. In the end, only patients with very similar CFI-S records are connected with a link, and they are located closer to each other in the graph. Graph neural networks (GNNs) are advanced neural network architectures developed based on graph theory concepts [17]. We also develop similarity network based GNNs to do suicidality prediction. Network Representation Link: https://github.com/cmxxx/SI.

## Results

### Traditional analyses

After collecting four-year follow-up data through electronic medical records chart review, analyses were performed using simple statistical tools.

**Fig. 1:**
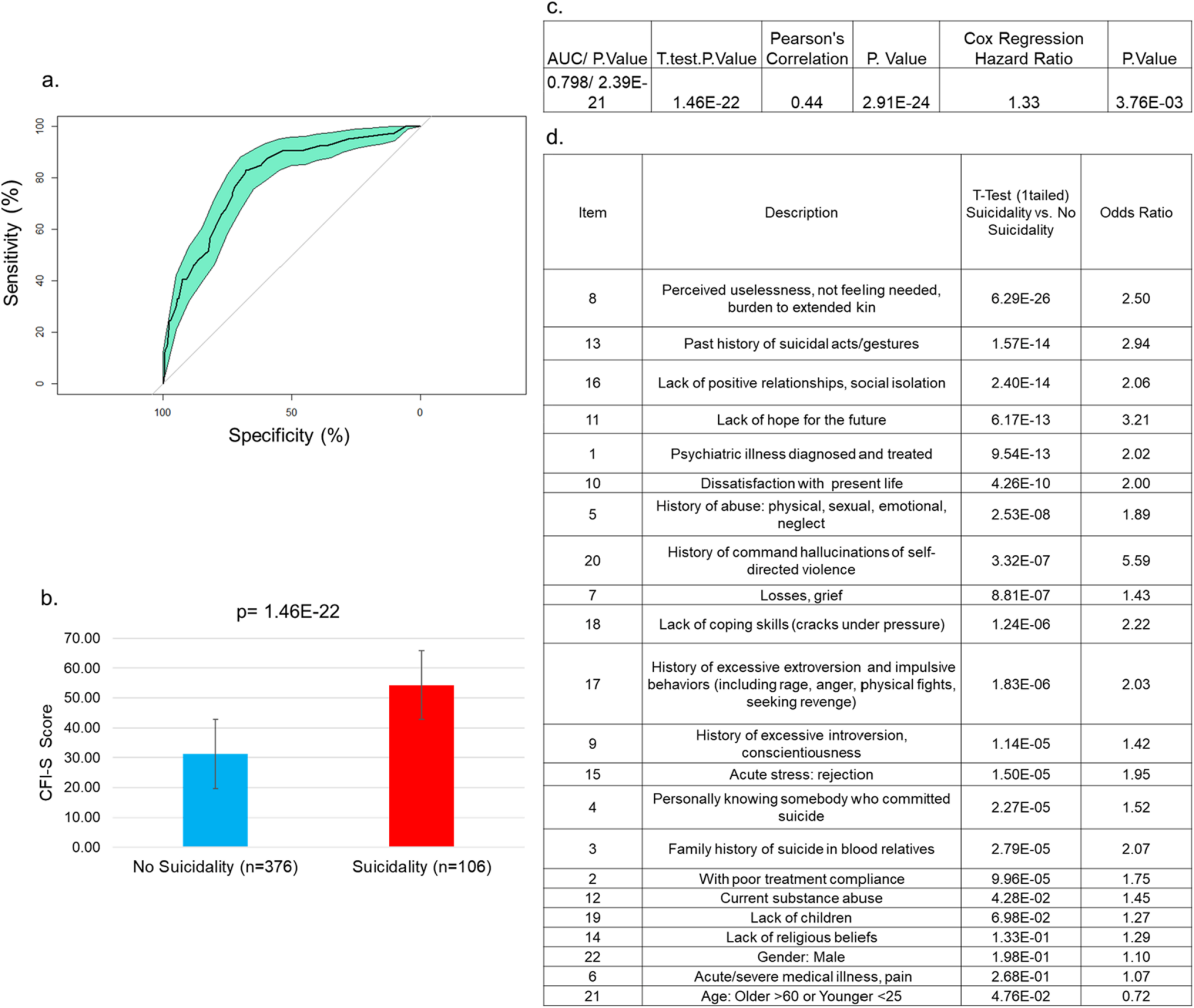
Traditional analyses. **a** ROC AUC **b** *T*-test. **c** Summary of results **d**. Individual items *T*-test

The overall CFI-S score was predictive of any future suicidality (ideation, planning, attempts, hospitalizations) with a ROC AUC of 0.798 and a *p*-value of 2.39 E−21 (Fig. 1a).

The average CFI-S score for those with future suicidality was 54 vs. 31 for those without future suicidality, with a *t*-test *p*-value of 1.46 E−22 (Fig. 1b).

We also examined the correlation of the CFI-S score with suicidality severity—suicidal ideation (SI) receiving a score of 1, suicide plan (SP) receiving a score of 2, suicide attempt (SA) receiving a score of 3, and hospitalization for suicidality receiving a score of 4. The Pearson’s correlation R-coefficient was 0.44, *p*-value of 2.91 E−24(Fig. 1c).

Additionally, a Cox regression was used to determine imminence of suicidality, producing a Hazard Ratio of 1.33 with a *p*-value of 7.53 E−03 and a one tailed *t*-test with a value of 3.76 E−03 (Fig. 1c).

A *t*-test was also performed for each individual CFI-S items between those with suicidality and those without (Fig. 1d). The top item (*p*-value 6.29 E−26, 12 orders of magnitude higher than the second best) was perceived uselessness (not needed, and/or feeling like a burden to kin). The next top items, in order, were past suicidality (1.57 E−14), social isolation (2.40 E−14). hopelessness (6.17 E−13), and past history of a mental health diagnoses (9.54 E−13).

### Machine learning analyses

Machine learning has the ability to extract more out of data, and it has been used for various medical diagnosis, such as tree-based models in PTSD assessment [18], naïve Bayes, random forest, and support vector machines in lung cancer prognosis [19], XGBoost for kidney disease diagnosis [20]. We developed a comprehensive machine learning framework for predicting future suicidality occurrence, severity, and imminence (see Tables 1, 2).

**Table 1:** Aggregate demographics

Analyses	Cohort	Number of participants	Gender	Ethnicity	Age mean (SD)
Traditional	No suicidality	376	Male 180 Female 195 Other 1	EA 192 AA 158 Hispanic 15 Asian 2 Other 9	44.6 (14.8)
	Suicidality	106	Male 55 Female 51	EA 56 AA 44 Hispanic 2 Other 2 American Indian 1 Asian 1	39.6 (13)
Machine learning	Discovery cohort	No suicidality 255 Suicidality 56	Male 128 Female 126 Other 1	EA 136 AA 106 Hispanic 10 Asian 2 Other 1	43.5 (14.8)
	Test cohort	227 No suicidality 50 suicidality	Male = 107 Female = 120	EA 112 AA 96 Other 9 Hispanic 7 American Indian 1 Asian 1 Other 1	43.5 (14.4)

**Table 2:** Confidence interval results of machine learning methods in suicidality prediction

Cohort	Methods	95% Confidence interval of evaluation metrics									
		Accuracy		Precision		Recall		F1 score		AUROC	
Discovery	NB	0.692	0.798	0.751	0.849	0.692	0.798	0.707	0.811	0.760	0.856
	XGB	0.777	0.871	0.773	0.867	0.777	0.871	0.774	0.868	0.758	0.851
	RF	0.866	0.938	0.866	0.938	0.866	0.938	0.860	0.934	0.831	0.913
	SVC	0.670	0.780	0.742	0.842	0.670	0.780	0.688	0.796	0.737	0.837
	DNN	0.755	0.853	0.802	0.890	0.755	0.853	0.766	0.862	0.792	0.882
Test	NB	0.678	0.792	0.744	0.848	0.678	0.792	0.698	0.810	0.764	0.866
	XGB	0.745	0.849	0.728	0.835	0.745	0.849	0.734	0.840	0.704	0.814
	RF	0.730	0.838	0.705	0.816	0.730	0.838	0.712	0.822	0.691	0.803
	SVC	0.769	0.869	0.751	0.855	0.769	0.869	0.739	0.846	0.681	0.795
	DNN	0.779	0.877	0.763	0.865	0.779	0.877	0.757	0.859	0.856	0.936

The future suicidality prediction is formulated as a binary classification problem. We developed a deep neural networks (DNN) framework, and compared it with other classical machine learning classifiers—native Bayes (NB), XGBoost (XGB), random forest (RF), support vector machines (SVM).

**Fig. 2:**
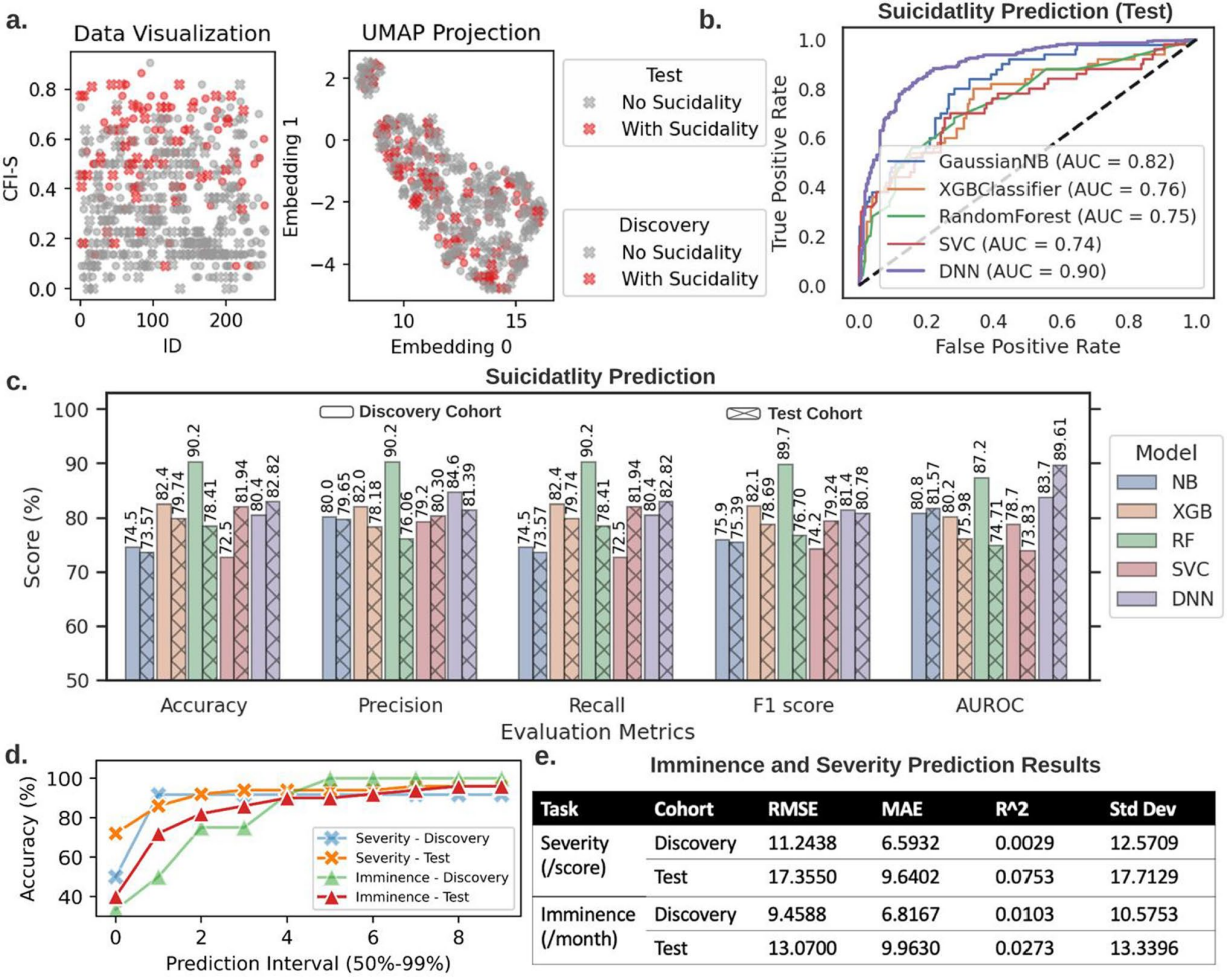
Machine learning analyses. **a** Visualization of the discovery (255 patients) and the validation (227 patients) cohort in the raw data and the uniform manifold approximation and projection (*UMAP*) space, respectively. The UMAP projection transforms the high-dimensional data into a 2D visualization and shows that the two suicidality classes (0 and 1) are overlapping by a large margin. This preliminary data inspection demonstrates the difficulty posed to the supervised machine learning tasks. **b** The receiver operating characteristic (ROC) curve comparison among several classifiers (i.e., naive Bayes (NB), XGBoost (XGB), random forest (RF), support vector machine (SVM), and deep neural network (DNN) classifier) for the suicidality classification. **c** The accuracy, precision, recall evaluation metrics, F1 score, and area under the receiver operating characteristic (AUROC) for the considered classifiers in the suicidality classification. The RF and DNN classifier emerge as the best model in discovery and test cohorts, respectively. **d** DNN accuracy increases for larger prediction intervals (PI) for the imminence and severity prediction on the discovery and test cohorts. **e** Since the imminence prediction and severity prediction represent regression problems, we report several standard evaluation metrics, such as the root mean square error (RMSE), mean average error (MAE), R-squared (R^2), and standard deviation. The usage of the discovery cohort in all experiments is the same as the training set in standard machine learning problems, and the test cohort can be seen as equivalent to the external test set

The receiver operating characteristic (ROC) curve, accuracy, precision, recall evaluation metrics, F1 score, and area under receiver operating characteristic (AUROC) results in Fig. 2b-c show that the constructed RF and DNN classifiers exhibit superior performance compared to the other classical machine learning classifiers for the discovery and test cohorts, respectively. For the results shown in this figure, we train and tune hyper parameters of our machine learning models with a discovery cohort, and then we get the test result by testing our models with an independent test cohort. Therefore, models that achieve good results in the test cohort are better than models that perform well in the discovery cohort. I.e., DNN achieves higher results in the test cohort, which demonstrates its generalization ability. The proposed DNN model is a complex and high-performance deep learning model, that takes CFI-S information as input and learns to utilize input data intelligently to achieve best performance possible within training time (see "Materials and methods" section, "Deep neural networks’ hyperparameters and training details" section for model details).

**Fig. 3:**
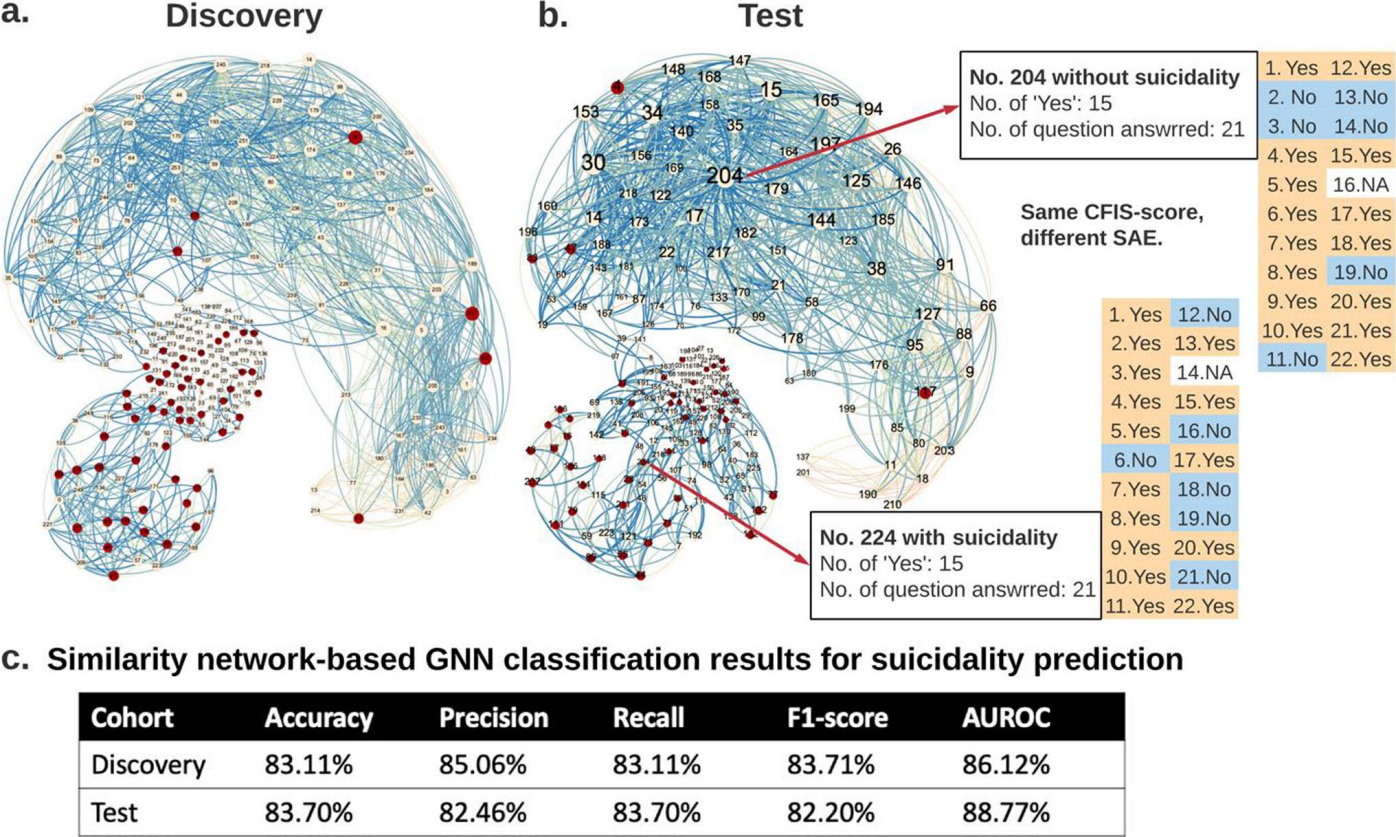
Similarity network classifier for visualizing patient’s risk. The discovery cohort (**a**) and test cohort (**b**) CFI-S-based similarity networks consist of nodes (patients) and weighted edges capturing the cosine similarity among pairs of patients’ CFI-S records. The edge color depends on the weight value. The node color indicates if a patient has suicidality. This topological representation of the CFI-S’s patients data shows that patients with suicidality (red nodes) are more clustered together towards the lower left part of the networks, while patients without suicidality (yellow nodes) are located mostly in the rest of the network. From the results shown in (**c**), we can see that similarity network-based GNN provides results that are comparable with DNN model

In addition to classical machine learning and deep neural network classifiers, we constructed patient similarity networks (see "Materials and methods" section, "Network representation" section for network representation details). With graph visualization shown in Fig. 3, we can locate and visualize a new patient in the graph based on collected CFI-S records, which is useful for potential early stage screening. Imagine a case where a patient takes 5 min and provides the CFI-S record, we can then compute the similarity between this CFI-S and all the other records we have in the system, then visualize the location of this patient in the graph. The graph has approximately 2 parts, the smaller area located in the lower left corner, which is a “high-risk” area, and the larger area located in the upper right corner of the graph is a “low-risk” area. With patients located in the graph, we provide a fast early-stage screening through graph neural network (GNN). GNN is an advanced graph based neural network model that works well on data that can be represented in graph or network. We formulate our GNN with this similarity network and provide a SI prediction. From the results shown in Fig. 3c, we can see that similarity network based GNN not only operates as an advanced classification model, but also provides explainability through visualization.

Different from predicting future suicidality occurrence, the severity and imminence predictions are formulated as regression problems. Severity represents the weighted score in relation to the severity of suicidality of a patient in a 4-year follow-up. Imminence refers to the time (month) elapsed between the CFI-S assessment and the first instance of suicidality of a patient. We used our DNN framework to investigate these two regression problems. Figure 2d, e summarize the prediction results of the proposed DNN model and other classical machine learning models (see also Supplementary Materials section X for details on the experimental setup and additional results.) The accuracy in Fig. 2d for the severity prediction and imminence prediction ranges between 85 to 100% and 90% to 96%, respectively, for increasing prediction intervals. The results for the test cohort are slightly lower than those in the discovery cohort. This demonstrates that the proposed deep learning framework can prove instrumental in suicide investigation, and may generalize well in external and future cohorts.

## Discussion

A simple, easy to administer, 22 items polyphenic risk score scale for suicidality, the CFI-S, which encompasses known risk factors but does not ask about suicidal ideation, was administered in an Emergency Department setting. The score was predictive of suicidality over the long-term, i.e., the ensuing 4 years of follow-up, with an AUC of 80% and a Hazard Ratio of 1.33. This simple tool, which on average took 5 min to administer, can be used in any setting, and provide a personalized mitigation plan by looking at the items that tested positive. Of note, the CFI- S does not ask about suicidal ideation, unlike the Beck Scale [21] or the Columbia Scale [22]. It is thus complementary to them. Patients do not always want to answer questions about suicidal ideation, for fear of being hospitalized. They might be less hesitant to answer the questions of an innocuous questionnaire like the CFI-S.

Machine learning approaches boosted the predictive ability of CFI-S up to 90%. By combining these analyses with a network science visualization, we developed a similarity network classifier for visualizing patient’s risk. Our CFI-S-based graph has two main components: a smaller area located in the lower left corner, which represents patients of “high-risk” suicidality, and a larger area located in the upper right corner of the graph, which represents patients with a “low-risk” of suicidality. This can be used for early-stage screening of suicidality risk, by showing where a new individual fits, based on the CFI-S risk score, compared with a well-studied normative cohort such as ours. For instance, one can exploit our framework for developing an AI system that can analyze the CFI-S answers of a patient during a short (5 min) interview, estimate the higher-order similarity scores between these newly recorded CFI-S scores and those in the cohort of patients prone to suicidality, visualize the position of the patient on the graph and determine a personalized strategy to mitigate suicide risk. Our proposed framework can be extended to encompass multiple data modalities (e.g., time taken to respond, hesitation to respond to specific questions, tendency to avoid some clear answers), in order to identify additional signatures of cognitive, physiological and behavioral nature that can help better predict suicidality.

Besides CFI-S, other types of information about the patients (e.g., age, ethnicity, career related metrics, social engagement) could be infused in the future into the machine learning framework to improve the model performance. For instance, the time spent on each CFI-S question, and facial expression images or videos, could be useful in inferring the emotion and trustworthiness of CFI-S answers. Genomic biomarker data, and other mental health phenotypic data, could also be integrated alongside the CFI-S, in a bio-psycho-social algorithm. This is the direction our future collaborative work is taking.

Lastly, it is particularly interesting, and actionable for preventive approaches, that feeling useless and socially isolated are top risk factors identified by our studies. The version of the instrument used at that time for this study had 22 items. We are developing now an expanded version that has 33 items, with more suicidality risk factors included. This version has not been yet been fully analyzed and compared to the original version, but may reveal in future studies additional actionable risk factors.

## Supplementary Information

Below is the link to the electronic supplementary material.

Supplementary file1 (DOCX 130 KB)

## Data Availability

The datasets generated during and/or analyzed during the current study are available from the corresponding authors on reasonable request.

## References

[1] Niculescu AB (2017). Precision medicine for suicidality: from universality to subtypes and personalization. Mol Psychiatry.

[2] Boggs JM (2018). General medical, mental health, and demographic risk factors associated with suicide by firearm compared with other means. Psychiatr Serv.

[3] Nock MK (2022). Prediction of suicide attempts using clinician assessment, patient self-report, and electronic health records. JAMA Netw Open.

[4] Brucker K (2019). Assessing risk of future suicidality in emergency department patients. Acad Emerg Med.

[5] Levey DF (2016). Towards understanding and predicting suicidality in women: biomarkers and clinical risk assessment. Mol Psychiatry.

[6] Niculescu AB (2015). Understanding and predicting suicidality using a combined genomic and clinical risk assessment approach. Mol Psychiatry.

[7] Oquendo MA, Baca-Garcia E, Mann JJ, Giner J (2008). Issues for DSM-V: suicidal behavior as a separate diagnosis on a separate axis. Am J Psychiatry.

[8] Domingos P, Pazzani M (1997). On the optimality of the simple Bayesian classifier under zero-one loss. Mach Learn.

[9] Chen T, Guestrin C. In: Proceedings of the 22nd acm sigkdd international conference on knowledge discovery and data mining. p. 785–794.

[10] Ho TK. In Proceedings of 3rd international conference on document analysis and recognition. In: IEEE. pp. 278–282.

[11] Cortes C, Vapnik V (1995). Support-vector networks. Mach Learn.

[12] LeCun Y, Bengio Y, Hinton G (2015). Deep learning. Nature.

[13] Glorot X, Bordes A, Bengio Y. JMLR Workshop and Conference Proceedings. In Proceedings of the fourteenth international conference on artificial intelligence and statistics. 2011. pp 315–323.

[14] Goodfellow I, Bengio Y, Courville A. Deep feed forward networks. Mit Press Essent. 2016. pp. 163–220.

[15] Kingma DP, Adam BJ. In International Conference for Learning Representations (San Diego, CA, 2014).

[16] Han J, Moraga C (1995). International workshop on artificial neural networks.

[17] Zhou, J. *et al.* Vol. 1 57–81 (Elsevier, 2020). https://www.sciencedirect.com/science/article/pii/S2666651021000012.

[18] Brenner LA (2021). Development and validation of computerized adaptive assessment tools for the measurement of posttraumatic stress disorder among US military veterans. JAMA Netw Open..

[19] Yu K-H (2016). Predicting non-small cell lung cancer prognosis by fully automated microscopic pathology image features. Nat Commun.

[20] Ogunleye A, Wang QG (2019). XGBoost model for chronic kidney disease diagnosis. IEEE.

[21] Baertschi M, Costanza A, Canuto A, Weber K (2019). The dimensionality of suicidal ideation and its clinical implications. Int J Methods Psychiatr Res.

[22] Posner K (2011). The Columbia-suicide severity rating scale: initial validity and internal consistency findings from three multisite studies with adolescents and adults. Am J Psychiatry.

