# Supplementary material for: Polyphenic risk score shows robust predictive ability for long-term future suicidality
Source: Discov Ment Health. 2022 Jun 13;2(1):13. doi: 10.1007/s44192-022-00016-z (PMC9192379; doi:10.1007/s44192-022-00016-z)
Supplement: Supplementary file 1 — Supplementary file1 (DOCX 130 KB) [file 44192_2022_16_MOESM1_ESM.docx]

**Supplementary Information**

**Convergent Functional Information for Suicidality (CFI-S) Scale**

©Niculescu 2015, 2016, 2017

Items are scored 1 for Yes, 0 for No. Total Score has a maximum possible of 22. Final Score is Total Score divided by number of items that were scored (as for some items information might not be available (NA) so they are not scored), and multiplied by 100.

| **Items** | **Yes** | **No** | **NA** |
| --- | --- | --- | --- |
| 1. **Psychiatric**   Ever had psychiatric illness diagnosed and treated |  |  |  |
| 1. **Compliance**   Ever had poor treatment compliance. |  |  |  |
| 1. **Familial**   Ever had family history of suicide attempts/completions in blood relatives |  |  |  |
| 1. **Example**   Ever personally know somebody who attempted/completed/ suicide |  |  |  |
| 1. **Abuse**   Ever had history of abuse growing up: physical, sexual, emotional, neglect, including bullying? |  |  |  |
| 1. **Medical**   Now or in the last 3 months have you had acute/severe medical illness, including acute pain (“I just can’t stand this pain anymore.”)? |  |  |  |
| 1. **Losses**   Now or in the last 3 months have you had losses, grief? |  |  |  |
| 1. **Useless**   Ever had feelings of uselessness, of not being needed, of being a burden to others? |  |  |  |
| 1. **Introverted**   Ever been excessively introverted, internally driven loner, overly conscientious, perfectionistic? Has it ever been hard for you to confide or seek help from others? |  |  |  |
| 1. **Unhappy**   Now or in the last 3 months have you been dissatisfied with your life? |  |  |  |
| 1. **Hopeless**   Now or in the last 3 months have you lacked hope about your future ? |  |  |  |
| 1. **Addiction**   Now or in the last 3 months have you abused alcohol or drugs? |  |  |  |
| 1. **History**   Ever had a past suicidal act, gesture or attempt? |  |  |  |
| 1. **Non-religious**   Now or in the last 3 months have you lacked religious (or spiritual) beliefs? |  |  |  |
| 1. **Rejection**   Now or in the last 3 months have you experienced separation, rejection by romantic interest, peers, friends? |  |  |  |
| 1. **Isolation**   Ever had lack of positive relationships, social isolation (externally driven loneliness, being shunned or ostracized by others)? |  |  |  |
| 1. **Impulsive**   Ever had periods of excessive extroversion and impulsive behaviors (including rage, anger, physical fights, school suspension, expulsions)? |  |  |  |
| 1. **Non-coping**   Ever had lack of coping skills when faced with stress? |  |  |  |
| 1. **Childless**   Now or in the last 3 months, are you childless? Or if you have children, is it true that you are not in touch /not close to them? Biological (1point), step (0.5 points) |  |  |  |
| 1. **Hallucinations**   Ever had command hallucinations of self-directed violence (voices telling you to harm yourself)? |  |  |  |
| 1. **Age**   Age: Older >60 or Younger <25 |  |  |  |
| 1. **Gender**   Gender: Male (1 point) or LGBTQ+ (0.5 points) |  |  |  |

***Niculescu AB**, Levey DF, Phalen PL, Le-Niculescu H, Dainton HD, Jain N, Belanger E, James A, George S, Weber H, Graham DL, Schweitzer R, Ladd TB, Learman R, Niculescu EM, Vanipenta NP, Khan FN, Mullen J, Shankar G, Cook S, Humbert C, Ballew A, Yard M, Gelbart T, Shekhar A, Schork NJ, Kurian SM, Sandusky GE, Salomon DR. [Understanding and predicting suicidality using a combined genomic and clinical risk assessment approach.](http://www.ncbi.nlm.nih.gov/pubmed/26283638) *Molecular Psychiatry*. 2015 Aug 18. doi: 10.1038/mp.2015.112. [Epub ahead of print].

Levey DF, Niculescu EM , Le-Niculescu H, Dainton HD , Phalen PL , Ladd TB _,_ Weber H, Belanger E, Graham DL, Khan FN, Vanipenta NP, Stage EC, Ballew A, Yard M, Gelbart T, Shekhar A, Schork NJ, Kurian SM, Sandusky GE, Salomon DR, **^*^Niculescu AB**. Towards understanding and predicting suicidality in women: biomarkers and clinical risk assessment. *Molecular Psychiatry* 2016 Jun;21(6):768-85. Epub 2016 Apr 5.

***Niculescu AB**, Le-Niculescu H. Dissecting Suicidality Using a Combined Genomic and Clinical Approach. *Neuropsychopharmacology* 2017 **42,** 360; doi:10.1038/npp.2016.228.

**Niculescu AB**, Le-Niculescu H, Levey DF, Phalen PL, Dainton HL, Roseberry K, Niculescu EM , Niezer JO, Williams A, Graham DL, Jones TJ, Venugopal V, Ballew A, Yard M, Gelbart T, Kurian SM, Shekhar A, Schork NJ, Sandusky GE, Salomon DR. Precision Medicine for Suicidality: from universality to subtypes and personalization. *Molecular Psychiatry* 2017. Sep;22(9):1250-1273. doi: 10.1038/mp.2017.128. Epub 2017 Aug 151 PMID: 28809398

Brucker KM, Duggan C Roseberry K, Le-Niculescu H,  ***Niculescu AB**,  *Kline JA.  Assessing Risk of Future Suicidality in Emergency Department Patients,  *Academic Emergency Medicine*, 2018  Oct 30. doi: 10.1111/acem.13562. [Epub ahead of print] PMID: 30375082

Table S1. Imminence prediction results using the suicide attempt as indicator.

| Task | Cohort | RMSE | MAE | R^2 | Std Dev |
| --- | --- | --- | --- | --- | --- |
| Severity (/score) | Discovery | 19.2158 | 14.4524 | -0.5869 | 24.8075 |
|  | Test | 16.7624 | 12.7645 | -0.4822 | 17.9198 |

**
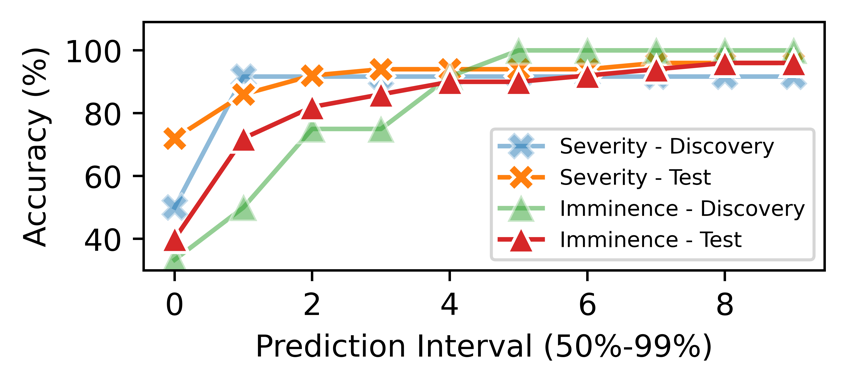
**

Figure S1. Accuracy results of imminence prediction using the suicide attempt as indicator.

# Evaluation metrics

## Suicidality classification

To better understand the evaluation metrics, we introduce the terminologies used in the evaluation metric calculation. Assume for a binary classification problem, there are two classes in the data, one is positive and the other is negative. Therefore, the number of real positive cases in the data is denoted as condition positive (P), and the number of real negative cases in the data is denoted as condition negative (N).

Once we have a classification model, we use the model to do prediction and evaluate the performance of this model. Among the predictions made by the classification model, a prediction that correctly indicates the presence or absence of a condition, is denoted as true positive (TP) or true negative (TN). On the contrary, a prediction that wrongly indicates the presence or absence of a condition is denoted as false positive (FP) or false negative (FN). By definition, we know that P=TP+FN, and N=TN+FP.

### *Recall / sensitivity / true positive rate (TPR)*

Recall, also known as sensitivity or TPR, represents how many relevant items are retrieved, and its mathematical formula reads: TPR=TP/P.

### *Precision / positive predictive value (PPV)*

Precision represents how many retrieved items are relevant, and it’s calculated as PPV=TP/(TP+FP).

### F1 score

F1 score is the harmonic mean of precision and recall, its mathematical formula is as follows: F1=2*PPV*TPR/(PPV+TPR).

### Accuracy

Accuracy is a statistical measure of how well a classification model predicts the condition, and its mathematical formula reads: ACC=(TP+TN)/(P+N).

### AUROC

A receiver operating characteristic curve, or ROC curve, is created by plotting the true positive rate (TPR) against the false positive rate (FPR) at various threshold settings. The true-positive rate is also known as sensitivity or recall. AUROC, i.e., the area under the ROC curve, is a metric frequently used to evaluate classifiers.

Confidence Interval (CI)

We calculated the CI for any evaluation metrics by p +/- Z*SE, where p is the evaluation metric, Z is the quantile in the standard normal distribution, and SE is standard error calculated as sqrt(p*(1-p)/N), where N is sample size. Commonly used confidence levels in practice are 90%, 95%, 98%, and 99%.

## Imminence and severity prediction

The evaluation metrics we used in this regression problem includes root mean squared error (RMSE), mean absolute error (MAE), R-squared, and standard deviation (STD).

### RMSE and MAE

The RMSE of an estimator is defined as the square root of the mean squared error (MSE), where MSE is the average of the squares of the errors. Similarly, MAE is the average of the absolute errors. Errors in regression problems are defined as the difference between the estimated values and the actual values.

### R-squared

R-squared, also known as the coefficient of determination, is a goodness-of-fit measure of regression models. Given a dataset has N samples, each of them is marked as y1,...,yN, and a fitted regression model generates N fitted values y1’,...,yN’. Then, R-squared is defined as: R^2 = 1-SSres/SStot, where the sum of squares of residuals reads: SSres = $\sum{(yi-yi’)}^{2}$, and the total sum of squares reads: SStot = $\sum{(yi-y\_bar)}^{2}$, where y_bar is the mean of the data.

### STD

The standard error of the regression (STD), represents the average distance that the observed values fall from the regression line. Its mathematical formula reads: STD = ${(SSres/(N-2))}^{0.5}$.
